# Reduction of tungiasis prevalence, intensity, and morbidity during a two-year long community-based tungiasis control project in a hyperendemic region in Karamoja, Uganda

**DOI:** 10.1371/journal.pntd.0013149

**Published:** 2025-06-05

**Authors:** Hannah McNeilly, Francis Mutebi, Felix Reichert, Marlene Thielecke, Mike L. Banalyaki, George M. Mukone, Rebecca Arono, Hermann Feldmeier

**Affiliations:** 1 Edinburgh Medical School: Deanery of Biomedical Sciences, The University of Edinburgh, United Kingdom; 2 School of Veterinary Medicine and Animal Resources, College of Veterinary Medicine, Animal Resources and Biosecurity, Makerere University, Kampala, Uganda; 3 Department of Infectious Disease Epidemiology, Robert Koch Institute, Berlin, Germany; 4 Charité Center for Global Health, Institute of International Health, Charité University Medicine Berlin, Germany; 5 Innovations for Tropical Disease Elimination (IFOTRODE), Mukono, Uganda; 6 Institute of Microbiology, Infectious Diseases and Immunology, Charité University Medicine Berlin, Germany; The University of Hong Kong, CHINA

## Abstract

Tungiasis is a widespread and debilitating zoonotic Neglected Tropical Disease (NTD). Manual extraction of the sand fleas with non-sterile sharp instruments is the most common but unsafe treatment method in affected communities. Topical application of a dimeticone oil formula (NYDA) has previously been shown to be a safe and effective method of killing embedded sand fleas. The objective of this study was to evaluate a two-year long humanitarian One Health tungiasis control project in 17 villages in Napak district, Karamoja region, Northeastern Uganda. The community-based intervention included quarterly systematic tungiasis detection and treatment with the dimeticone oil formula in residents and domestic animals in combination with community health promotion. In each of the eight quarterly tungiasis diagnosis and treatment rounds, between 3,674 and 5,155 residents were examined (coverage 73.6-89.9%). Overall, 12,540 tungiasis cases among residents were diagnosed and treated and 16 community dialogue meetings were held. Tungiasis prevalence among residents decreased from 62.8% to 5.7% in the two-year study period. While at baseline tungiasis was most prevalent in children and the elderly, at the end elderly women were the single most affected group. The prevalence of tungiasis-related walking difficulties in the community decreased from 11.5% to 0.5%, and pain and itching were greatly reduced. The number of animals present in the villages was low (between 79 and 414 per treatment round) and the prevalence of tungiasis in animals dropped from 14.2% to 0% throughout the two-year project. This implementation study shows that regular community-based treatment of tungiasis cases among humans and animals with dimeticone oil formula, combined with community engagement and health promotion, can reduce tungiasis prevalence and morbidity to very low levels within two years, even in a hyperendemic area where people live in extreme poverty.

## Author summary

Sand flea disease (tungiasis) is a common poverty-associated disease in many tropical countries that affects humans and animals. It causes pain, itching, and walking difficulties and can lead to social isolation and life-threatening bacterial or viral infections. In absence of effective treatment, local communities commonly resort to the dangerous and painful practice of removing sand fleas with sharp objects like pins or thorns. Small studies have shown that applying a mixture of dimeticone oils (NYDA) is a simple and effective treatment. To demonstrate the real-life effects of a comprehensive control strategy for tungiasis, we evaluated a two-year long tungiasis control project in 17 villages in the rural Napak district, Karamoja region, Northeastern Uganda. This included regular detection of cases of sand flea disease among village residents and domestic animals, case treatment with the dimeticone oils, as well as conducting meetings with the residents to provide information about sand flea disease, its treatment and prevention. Throughout the study, the occurrence of sand flea disease in the study area decreased from 62.8% to 5.7% among residents and from 14.2% to 0% in animals. Walking difficulties due to sand fleas among residents dropped from 11.5% to 0.5%, and pain and itching were also greatly reduced. This study shows that the regular treatment of sand flea disease in humans and domestic animals with the dimeticone oil mixture, together with community engagement regarding sand flea control, can effectively reduce the burden of the disease to very low levels within two years, even in an area where sand fleas are widespread.

## Introduction

Tungiasis is a neglected tropical disease (NTD) caused by the female sand flea, *Tunga penetrans*, which burrows into the skin of humans and other mammalian species, where it causes inflammation for four to six weeks, before it dies *in situ* [1]. Off-host adult sand fleas are up to 1mm long [2] but when embedded in the skin, the female’s abdomen grows up to 1 cm in diameter within a week [3]. While embedded, the female sand flea feeds on blood and expels eggs through its posterior abdomen [1–4]. Most commonly, tungiasis is found in the skin of the feet, although ectopic sites like knees, hands, and buttocks can also be affected [5,6].

Tungiasis is prevalent among resource-poor populations in sub-Saharan Africa and Latin America, particularly during the dry season [7,8]. Typically, children of primary school age and the elderly are most affected [5,9,10]. In sub-Saharan Africa, very high prevalences have been documented, with up to 45% in rural communities and 59% among children [11,12]. In Uganda, a study found a prevalence of 23% in 12 villages in Mayuge district (Eastern region) [13]. Recently, our study team reported an overall tungiasis prevalence of 63% among 17 villages in Napak district, Northeastern Uganda [10].

Tungiasis causes considerable suffering due to pain and itching, sleeping problems, deformities and loss of nails and toes, walking difficulties, and potentially life-threatening secondary bacterial infections, such as tetanus or septicaemia [14–17]. Like other skin NTDs, tungiasis is a highly stigmatised condition that frequently leads to social isolation [15,18–21], which – coupled with physical impairment – traps patients and communities in a cycle of poverty [21–23]. In places where tungiasis is endemic, extremely severe and debilitating cases with hundreds of sand fleas may occur [22,24–26].

In affected communities, the most commonly used control practice is manual extraction of sand fleas from the skin by use of non-sterile sharp instruments (such as thorns, pins, needles, razor blades, or sharpened sticks) which are frequently shared within households and at community level [18,27–30]. This procedure intensifies the inflammation and causes significant pain, bleeding, open wounds, and a high risk of bacterial and viral infections [16,17,31]. Children in whom manual extraction of fleas was done repeatedly are prone to psychological trauma (H. Feldmeier and G.M. Mukone, personal observation 2013). Other local treatment attempts include the application of hazardous substances like motor oil, tobacco, naphthalene, and kerosene [18,29,30,32].

Systematic reviews of the efficacy of different oral and topical tungiasis treatments indicate that currently topical application of dimeticone oils (NYDA) is the most effective and safe treatment against tungiasis [33,34]. Dimeticone oils are silicone oils with high creeping properties due to their low surface tension. NYDA is a formula of two dimeticone oils with different viscosities, which has been established as a non-toxic and effective topical treatment for head lice in many countries [35,36]. Due to its purely physical mode of action it is registered as a medical device, and there is no risk of resistance developing. This dimeticone oil formula has been demonstrated to be highly effective against tungiasis in randomized controlled proof-of-principle trials in Kenya and Uganda [27,37]. Compared with topical treatment with potassium permanganate (KMnO4), the dimeticone oil formula has been shown to be more effective [27]. A study from Kenya showed that topical application of neem oil mixed with coconut oil also has a therapeutic effect [38], although dimeticone oil formula is more effective and requires less frequent application [27]. Applying a few drops of the dimeticone oil formula onto tungiasis lesions is a sufficient, safe, and easy treatment, and can be carried out by community members or patients themselves [27,37].

The dimeticone oil formula seals off the ‘achilles heel’ of the embedded female sand flea: a small opening (≈200µm) of the rear abdominal cone where the respiratory tract, the excretory system, and the genital tract remain in contact with the environment [1,27,35]. As a result, the embedded sand flea dies, inflammation decreases, and the sand flea carcass is eliminated by skin tissue repair mechanisms before reepithelialisation ensues [1,27,35]. A case series from Colombia and our longitudinal study in Northeast Uganda demonstrated that the dimeticone oil formula can effectively resolve even extremely severe tungiasis infections with hundreds of lesions [22,24]. When applied regularly, the dimeticone oil formula can result in the interruption of the transmission cycle [39], because less and less eggs will be expelled for the off-host life cycle phase to continue.

As tungiasis is a poverty-associated zoonosis, its control requires a comprehensive One Health approach including treatment as well as socio-environmental measures [4,40,41]. Animal and human tungiasis are closely associated. Pigs, dogs, cats, goats, and peri-domiciliary rodents are relevant animal reservoirs for *T. penetrans* [41–43]. Risk factor analyses in Kenya, Nigeria, Uganda, and Brazil showed that the main risk factors for tungiasis infection are low economic status, poor housing conditions, natural sand or earthen floors, dirty and cracked floors, limited access to water, washing the feet without soap, and presence of animals on the compound [9,13,44–47]. A study from Kenya showed that tungiasis prevalence could be sustainably reduced through environmental preventive measures [40]. The socio-environmental factors thus need to be addressed to enable effective and sustainable success of tungiasis control efforts [33,40]. In addition, a dialogue with the local communities and stakeholders is necessary to ensure that the strategy is appropriate and acceptable for the population and that it adequately responds to local needs and perspectives [29,39,48].

The objective of the present study was to evaluate a large community-based One Health tungiasis control project in a hyperendemic setting in Uganda.

## Methods

### Ethics statement

The study was scruitinized for sensitivity to local needs and sociocultural values by Napak District Local Government who provided administrative clearance of the project (Ref. CR/205/1). Ethical approval was obtained from the Vector Control Division of the Ministry of Health Ethical Committee, Uganda (VCDREC 112/UG-REC-018) and the study was accredited by the Uganda National Council of Science and Technology (HS2623) before it commenced. Written consent was obtained from all adult participants (over 18 years) and verbal assent was obtained from children after their parent/guardian had given written consent for their participation. Privacy was observed by examining or interviewing all participants in a location of their choice within or near their respective homesteads with or without other people present as they wished.

### Study design overview

This is an implementation study about a two year-long humanitarian community-based tungiasis control project in 17 villages in Ngoleriet sub county, Napak district, Karamoja, Northeastern Uganda. The humanitarian project was conducted by IFOTRODE (Innovations for Tropical Disease Elimination), a non-governmental organisation dedicated to NTD control. It involved (a) eight quarterly community-wide systematic rounds (periods) of diagnosis and treatment of humans and domestic animals with the dimeticone oil formula NYDA, (b) inter-round tungiasis treatment of self-reported cases carried out by community health workers, and (c) 16 community dialogue meetings in year 1 and continuous door to door health promotion during the regular diagnosis and treatment rounds.

Tungiasis prevalence among residents in the study area was the primary outcome measure monitored throughout the two-year study period. Secondary outcomes were tungiasis intensity and morbidity among recorded cases as well as tungiasis prevalence among domestic animals and in visitors to the study area.

For ethical reasons, this implementation study does not include a control group. Data was collected via mobile tools during each of the eight diagnosis and treatment rounds. Data collection took place from February 2021 to December 2022.

### Study area and study population

**Fig 1:**
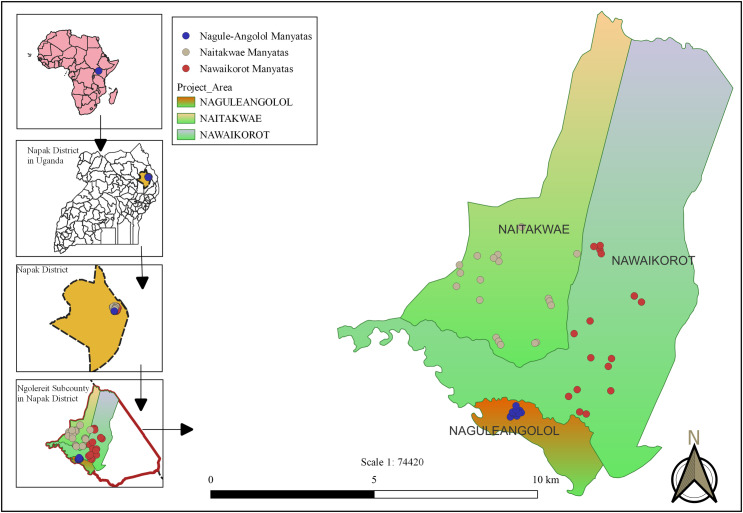
Map showing the location of the manyatas in the three parishes; as previously published [10].

We included the population of three out of the five parishes in Ngoleriet sub-county, Napak district, Karamoja region, in this study (Fig 1). Karamoja region consists of nine districts and is one of the most impoverished regions of Uganda [49]. The three parishes in Ngoleriet sub county, Napak district, were chosen, because in 2019, the District Health Office communicated to IFOTRODE that tungiasis was highly prevalent there, contributing to the humanitarian crisis in Karamoja, which is characterized by extreme poverty and constant food shortage [49]. This was confirmed in November 2020 when we used a previously tested rapid assessment method [50] in 11 villages in the study area, which showed a prevalence of 68.5% among 666 examined individuals [10].

The study area was composed of 17 villages. The village inhabitants lived in 52 *manyatas*, groups of houses and compounds that are surrounded by shared high fences made of sticks and thorny hedges to deter wild animals and intruders. Within the *manyatas*, households are further separated from each other by smaller aggregates of sticks.

At the beginning of the humanitarian project, during case detection and treatment round one (1^st^ February to 18^th^ March 2021), a population of 5,482 individuals (3,273 females and 2,209 males) lived in 1,334 households of varying sizes (mean = 4 people, range = 1–15) [10].

As the inhabitants of the study area have a semi-nomadic lifestyle, men and boys frequently leave the villages for several weeks or months to herd livestock. This led to a fluctuating population throughout the study period. Livestock that might act as reservoirs, such as goats, sheep, and cattle, were rarely present in the villages.

Table 1 shows the numbers of villages, *manyatas*, and residents of the three parishes during the study period. A detailed description of the study population at baseline can be found elsewhere [10].

**Table 1:** Population overview of the three study parishes in Ngoleriet sub-county. The presented population numbers include temporarily absent residents.

Parish	Number of villages	Number of *manyatas*	Mean population in 2021 and 2022 (range)
Naitakwe	7	23	1,904 (1,196 - 2,075)
Nawaikorot	6	17	1,779 (1,196 - 2,123)
Nagule Angolol	4	12	1,655 (1,368 - 1,852)
TOTAL	17	52	5,346 (4,043 - 5,799)

**Fig 2:**
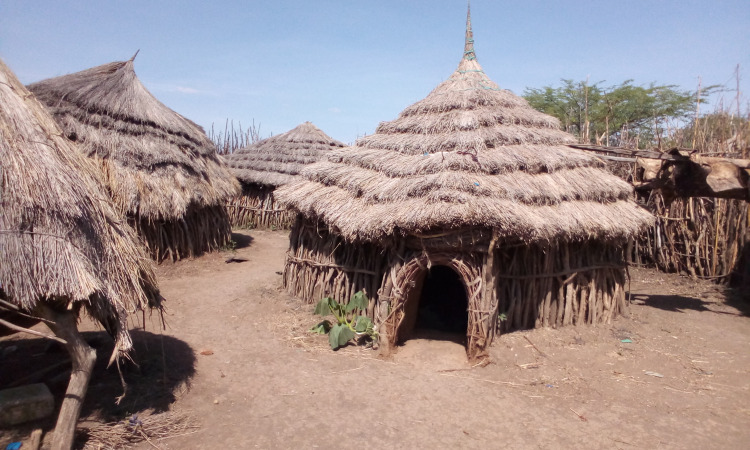
A typical homestead in a manyata in the study area. Photo: F. Mutebi 2022.

Living conditions in the study area were poor. Houses were made of sticks and mud, with grass roofs and earthen floors (Fig 2). The villages had no electricity, and water sources, such as bore holes, were frequently dysfunctional or located far away. The distance to the nearest functioning water source from each *manyata* ranged between 0 and 2.5km, with a median distance of 0.5km.

Compounds were often littered with plant material, such as sunflower, maize, and sorghum husks and household refuse. The floors of the houses were frequently dusty and dirty, thus providing favorable conditions for the completion of the off-host cycle of sand fleas [1,4]. Hunger and malnourishment were common, and in most households, people only had one meal per day [10]. Livestock rearing (mainly cattle, goats, and sheep), and subsistence crop farming (mainly sorghum, sunflower, and maize) were the mainstay for livelihoods. During our study, most animals were kept far away from villages, as cattle raids were common in the area [10]. Herding of animals was mostly done by older boys and young men, and crop farming and marketing of agricultural produce was mainly carried out by women and girls. Cats and dogs were kept in small numbers in the villages. They were usually not allowed to enter the houses and roamed freely, and in most cases accompanied herdsmen while grazing animals. The few cattle, sheep, and goats that remained in the villages were kept in small communal enclosures.

At the time of data collection, Ngoleriet sub-county had two health centres in Nawaikorot, which were run by nurses and auxiliary medical staff and offered free health services. However, the health centers were almost inaccessible to the population of the study area, as they were located far away from most villages, were frequently shut, and only had access to few drugs. The health centers did not offer tungiasis treatment. In the study area, tungiasis was habitually managed by residents by way of manual extraction with non-sterile, sharp instruments like thorns, pins and needles, which were often shared, and application of different substances, including toxic ones like tobacco, kerosene and used motor oil [18,29].

### Humanitarian tungiasis project

#### Recruitment and training of village tungiasis health workers (VTHWs).

At the start of the project, IFOTRODE recruited and trained eight local village tungiasis health workers (VTHWs) in each of the three study parishes, resulting in a team of 24 VTHWs (15 women and 9 men). The VTHWs were residents of Ngoleriet sub county and were fluent in the local language Ngakarimojong and English. Training was delivered locally by IFOTRODE’s physician (G.M. Mukone) and the project veterinarian (F. Mutebi) over a course of seven full days. It covered physical examination for tungiasis in humans and animals; correct application of dimeticone oil (NYDA) for treatment; how to advise local people on recognising, treating, and preventing tungiasis; application of questionnaires; data entry via the mobile app; and ethical aspects of data collection. The already existing government-established village health teams (VHTs) could not be employed for data collection as their team members did not speak English, and most could not read and write. However, the VHTs supported the VTHWs as field guides and community mobilisers, together with the Local Council 1 (LC1) village leaders. Field activities were supervised by two social workers and a study nurse.

#### Case finding and data collection.

During the two years of the humanitarian tungiasis control project, eight systematic rounds of case detection and treatment for tungiasis were conducted in the study area, approximately every three months. The project team visited the study villages between 1p.m. and 5p.m. (Monday to Friday), when most people were present in or near their houses, and between 9a.m. and 2p.m. on weekends. Residents were approached door-to-door by the VTHWs who explained the procedures and the study and asked for consent. If a resident could not be found at home or nearby, the VTHWs would revisit their home twice over the following days. People who only stayed in the area temporarily as visitors were also included. We defined visitors as people present during the tungiasis detection and treatment rounds who had been staying in the area for less than three months and who did not have a permanent home there.

**Fig 3:**
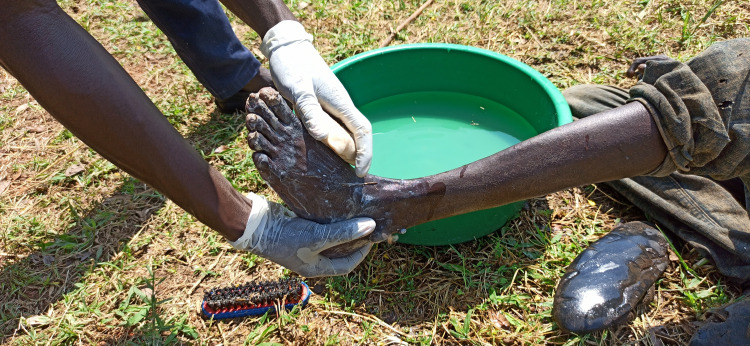
Washing of the feet before examination, carried out by the VTHWs. Photo: M.L. Banalyaki 2022.

Before examination, VTHWs washed the participants’ feet, legs, and hands with water, soap, and a brush to improve the visibility of skin lesions (Fig 3). They then inspected the feet, legs and hands for tungiasis lesions. Participants were asked if they had sand fleas (locally called *ngidud*) in other body areas, and if this was the case and the participant gave consent, these areas were also inspected. Domestic animals were examined, after adequate restraint, by inspection and palpation while parting the fur, with focus on the digits and legs, and the trunk and head were examined too [10,41].

When tungiasis was diagnosed, the VTHWs counted the sand flea lesions and classified them based on their morphology [1] (Table 2). Tungiasis prevalences and intensities were determined based on the presence and number of any type of tungiasis lesions (viable, dead, or manipulated), respectively. Mild tungiasis cases were defined as the presence of 1–5 tungiasis lesions, moderate cases as 6–30 lesions, and severe cases as > 30 lesions [44]. Some study participants presented with extremely high numbers of sand flea lesions (100 or more), and were categorised as ‘very severe cases’.

**Table 2:** Classification of tungiasis lesions [1].

Type of tungiasis lesion	Presentation
Viable	Dark brown to black spots in the centre of a hyperaemic rim or yellow-to-white nodular lesions of 2–12 mm in diameter
Dead	Raised circular brown to black patches or shallow circular skin craters with necrotic edges
Manipulated	Skin sores from which embedded sand fleas have been completely or partially removed with a sharp instrument

Data on the presence of tungiasis, number and type of lesions, and tungiasis-related pain, itching, and mobility impairment was recorded by the 24 VTHWs during the case detection and treatment rounds, under supervision of the social workers and the study nurse. Individuals with tungiasis were asked to classify their pain and itching as ‘very much’, ‘quite a lot’, ‘a little’, and ‘not at all’ and if they had any tungiasis-related mobility restrictions. As these symptoms could not be reliably established in animals, they were only recorded in people with tungiasis. The VTHWs communicated with the participants in Ngakarimojong language and recorded their responses in English. Mobile phones were used for data collection with ODK (Open Data Kit) Collect, an open-source digital android app. Entered data was double-checked by the project data manager (R. Arono). Additional inter-round treatments in the villages were carried out by the VTHWs, recorded on paper, and were subsequently digitized.

#### Treatment for tungiasis.

**Fig 4:**
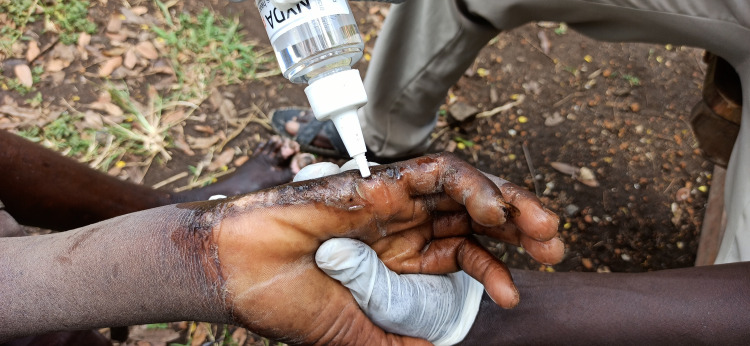
A VTHW applies dimeticone oil formula to sand flea lesions on a hand. Photo: M.L. Banalyaki 2022.

Diagnosed cases of tungiasis were immediately treated by topical application of the dimeticone oil formula (NYDA) (Fig 4). The VTHWs applied two to three drops to single lesions in mild cases and wetted larger skin areas or the whole foot in moderate, severe, and very severe infections. Mild and moderate cases were treated once only. In cases of severe and very severe tungiasis infection, topical application of the dimeticone oil formula was repeated every other day until no more viable embedded sand fleas could be detected.

In between the systematic case detection rounds, community members were encouraged to contact their VHT if they had new tungiasis lesions and seek inter-round treatment from the local VTHWs who were equipped with dimeticone oil formula in 100ml bottles. Infected animals were treated in the same way as humans.

#### Community engagement on village level.

During case detection and treatment rounds, the project team encouraged community members to wash their feet with water and soap daily, to keep house floors and compounds clean, and to regularly use the traditional method of smearing house floors with a mixture of cow dung and clay to smoothen the floor. Smearing house floors aimed at keeping tungiasis prevalence low in the long-term by minimising cracks where the off-host life cycle of sand fleas is completed. It is a widely used practice in rural communities in Uganda to make homes look more beautiful. Due to personnel constraints and to avoid intrusion, it was not followed up systematically whether individual households followed the advice given by the VTHWs.

In addition, 16 community dialogue meetings were held by members of the project team in strategically placed, easily accessible, open-air locations in the study area. All community members had the opportunity of attending at least one meeting without having to walk long distances. The community meetings took place between 23^rd^ September and 1^st^ October 2021, at the end of case detection and treatment round three. The aims of the community dialogue meetings were to receive feedback from community members about the project, identify local needs and opportunities regarding tungiasis control, and inform the communities in a comprehensive way about the disease and how to prevent and to treat it. In total, 803 adults and even more children (number not recorded) participated in the 16 meetings.

Following discussions at the community dialogue meetings, the IFOTRODE project team organized “tungiasis prevention weeks” in each of the villages to support messages on environmental prevention measures in practice. The “tungiasis prevention weeks” took place during case detection and treatment rounds 6, 7, and 8, and were supported by the VHTs, LC1 leaders, and sub-county technical staff and public servants. During the “prevention weeks”, village members swept their compounds and houses, eliminated organic matter from their surroundings, and smeared their floors with cow dung and clay to interrupt the off-host cycle. The labor and the materials for this activity were provided by the community members, and they were not supervised while implementing it. IFOTRODE supported elderly community members in these activities by fetching water for them using motorcycles.

#### Sustainability.

After completion of the two-year tungiasis project and data collection for this study, IFOTRODE implemented measures to ensure sustainability of the success of the control project (phase 2). Information posters and flip charts in English and the local language Ngakarimojong as well as booklets for health workers in English were developed by IFOTRODE in collaboration with the Napak District Health Office and the Ministry of Health. These contain health promotion messages regarding diagnosis, prevention and treatment of tungiasis and were distributed to villages, schools, health units, district and sub county offices in Napak District. Additionally, refresher training was organized for the VTHWs and the VHTs in the 17 villages of the study area to diagnose and treat tungiasis and to promote preventive measures. IFOTRODE provided VHTs with NYDA, soap, scrubbing brushes, towels, and gloves for two years after the systematic treatment rounds ended, and appealed to the Ministry of Health to continue to supply NYDA to the area thereafter. Moreover, IFOTRODE has established tungiasis control measures in 18 primary schools in Napak District by educating teachers and pupils about tungiasis prevention, diagnosis, and treatment. Data from phase 2 of the project is not presented in this paper.

Based on the success of the humanitarian tungiasis control project, in 2023 the Ugandan Ministry of Health added the dimeticone oil formula NYDA to the list of essential medicines [51] and developed a national guideline for tungiasis treatment [52] in partnership with IFOTRODE.

### Data analysis

Data analysis was carried out using STATA version 17 and R version 4.1.2 [53]. Due to the fluctuating population, this study was analysed as a repeated cross-sectional study. For each data collection time point, prevalence and 95% confidence intervals (CI) were calculated with the Wilson method. We calculated relative and absolute reduction of prevalence and tested if the prevalence between treatment rounds is equal using Pearson’s chi-squared test statistic with a 5% significance level. For intensity of infection (number of sand flea lesions per person), median and interquartile range (IQR) are presented. Relative reduction of the median and total number of lesions, the number of very severe cases as well as of cases with walking difficulties was calculated. Results refer to the residents in the study area, unless it is clearly stated that they refer to visitors or animals.

## Results

### Coverage

Coverage of the villages’ population during case detection and treatment rounds varied between 73.6% in round one and 89.9% in round 8 (Table 3). Less than 1% declined participation, but between 796 and 1,440 (average = 1,028.4) village residents were not available due to temporary absence during the respective rounds. The temporarily absent residents were mostly younger men herding animals in faraway pastures, boarding students, and women growing crops in other parts of the district and beyond. Overall, 35,940 examinations in 6,558 residents were carried out.

**Table 3:** Coverage of residents in the study area in the case detection and treatment rounds 2021-2022.

	Year 1 (2021)				Year 2 (2022)			
**Round**	**1**	**2**	**3**	**4**	**5**	**6**	**7**	**8**
Time period	1 Feb - 18 Mar	28 Apr - 2 July	16 Aug - 2 Oct	17 Nov -20 Dec	24 Jan - 14 Mar	28 Apr - 9 June	8 Aug - 2 Oct	3 Nov - 16 Dec
Residents^a^	5482	5379	5604	5591	4902	5716	5799	5733
Temporarily absent^b^	1440	1072	1061	1130	1226	796	924	578
Number of people examined (%)	4035 (73.6%)	4299 (79.9%)	4536 (80.9%)	4453 (79.7%)	3674 (74.9%)	4915 (86.0%)	4873 (84.0%)	5155 (89.9%)
Did not give consent	7	8	7	8	2	5	2	0

^a^ Residents were defined as people with a permanent home in the study area.

^b^ Temporarily absent residents had a permanent home in the study area but were not present during the treatment round, usually because they were herding animals or gardening far away from the village.

### Prevalence

**Fig 5:**
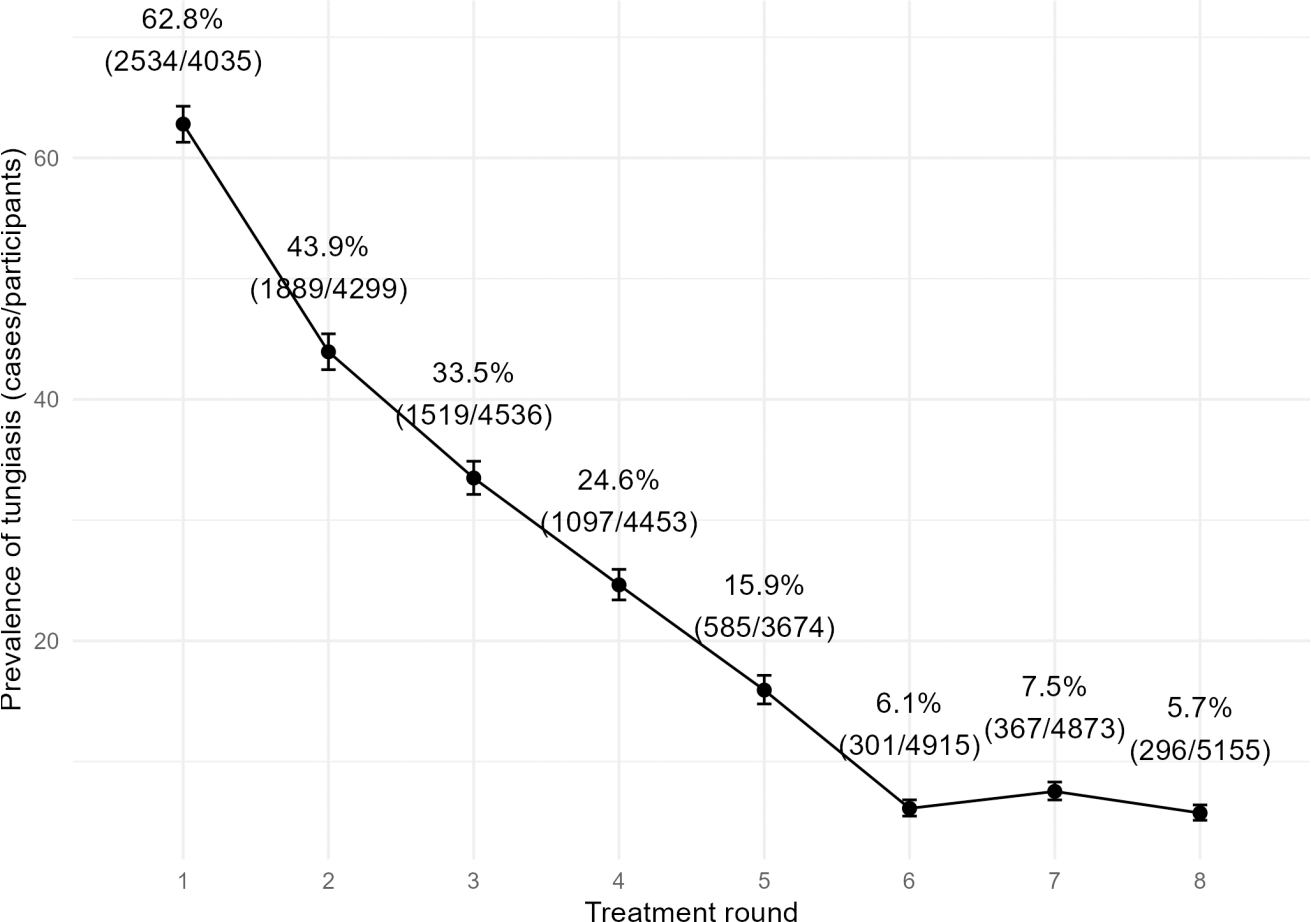
Change in tungiasis prevalence among residents examined throughout the tungiasis project. See Table 3 for start and end dates of the eight treatment rounds.

At baseline in February/March 2021 (detection and treatment round 1), tungiasis prevalence among the examined study participants was 62.8% [95% CI 61.3-64.3%] (n = 2,534 out of 4,035). Over the following seven rounds of screening and treatment, the prevalence of tungiasis in the study area continually decreased to 5.7% [95% CI 5.1-6.4%] (n = 296 out of 5,155) at the end of the study period (Fig 5). This represents a relative reduction of 90.9%.

There was a significant reduction of tungiasis prevalence compared to the previous treatment round in rounds 2–6 (p < 0.01). The biggest absolute reduction could be seen between rounds 1 and 2, when the prevalence in the study area dropped by 18.9 percentage points from 62.8% to 43.9% (Fig 5). It stabilized at a low level in rounds 6, 7 and 8.

**Fig 6:**
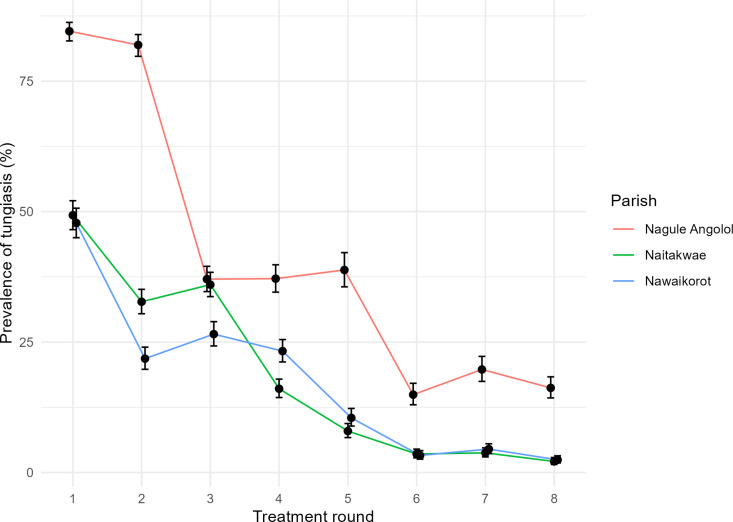
Reduction of tungiasis prevalence in the three study parishes.

The reduction in prevalence varied between the three study parishes (Fig 6). Nagule Angolol parish was by far the most affected, with a baseline tungiasis prevalence of 84.6% [95% CI 82.7-86.3] (n = 1,349/1,595) in round 1 and an endpoint prevalence of 16.2% [95% CI 14.3-18.3] (n = 207/1,276) in round 8. Naitakwe and Nawaikorot parishes had lower baseline prevalences of 49.3% [95% CI 46.5-52.1] (n = 615/1,247) and 47.8% [95% CI 45.0-50.7] (n = 571/1,193), respectively, and endpoint prevalences of 2.1% [95% CI 1.6-2.9] (n = 40/1,875) and 2.5% [95% CI 1.9-3.2] (n = 49/2,004), respectively. The absolute reduction of prevalence was thus higher in Nagule Angolol than in Naitakwe and Nawaikorot (68.4%, 47.2%, and 45.3%, respectively), while the relative reduction of prevalence was lower in Nagule Angolol compared to Naitakwe and Nawaikorot (80.8%, 95.8%, and 94.8%, respectively).

**Fig 7:**
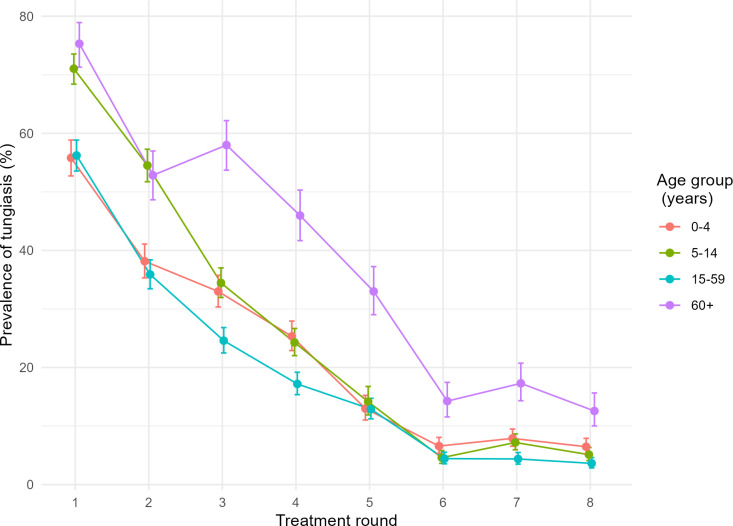
Tungiasis prevalence stratified by age group per treatment round.

Although baseline tungiasis prevalence was higher in boys and men (65.5% [95% CI 63.0-67.9]) than in girls and women (61.3% [95% CI 59.4-63.1]; p = 0.004), there was no relevant overall difference between sexes in the following rounds. Stratified by age, at baseline tungiasis was most prevalent in 5–14-year-olds (71.0% [95% CI 68.4-73.6]) and participants of 60 years and older (75.3% [95% CI 71.3-78.9]). All age groups saw a steep decline of tungiasis after round 1 (Fig 7). However, from round 3 on, the decrease in tungiasis among the elderly (≥ 60 years) lagged behind the other age groups (Fig 7).

Stratification by age and gender showed that this trend was driven by higher prevalences among elderly women (S1 Fig). At the end of the study, tungiasis prevalence among women ≥ 60 years remained significantly higher than among men ≥ 60 years (14.9% v. 5.3%, respectively) and any other demographic group (S1 Fig).

During the project period, 12,540 cases of tungiasis were diagnosed and treated with the dimeticone oil formula (Table 4). Most treatments took place during the systematic rounds (66.5%), though the proportion of inter-round treatments differed between the three study parishes (Table 4).

**Table 4:** Number of treatments carried out during the tungiasis control project according to parish.

Treatment	Number of tungiasis treatments			
	Nagule Angolol	Naitakwe	Nawaikorot	Total
Systematic treatment rounds	4,367	2,058	1,917	8,342
Inter-round treatments	1,401	1,217	1,580	4,198
Total	5,768	3,275	3,497	**12,540**

### Intensity of infection

**Fig 8:**
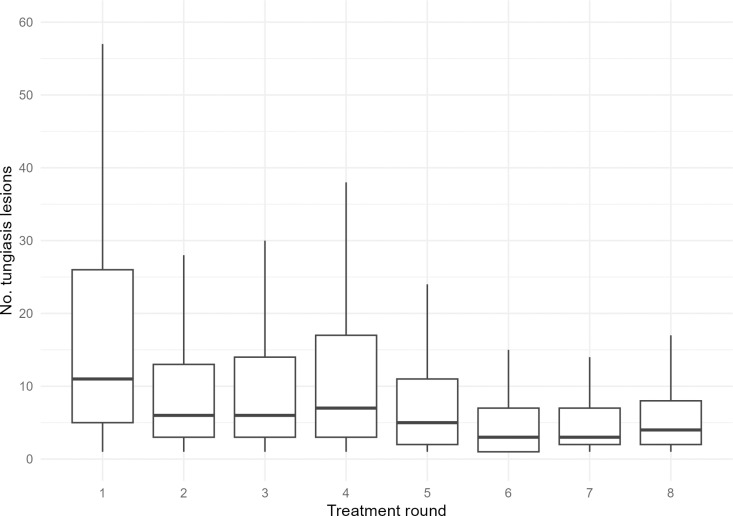
Number of tungiasis lesions in affected individuals (intensity) over time. The hinges correspond to the first and third quartiles, the whiskers extend to the smallest/highest value at most 1.5*IQR of the hinge. Outliers are not displayed.

In addition to the decreased prevalence, there was a significant reduction in intensity of tungiasis infections over the study period. The reduction was greatest after the first treatment round, when the median number of lesions (viable, dead, and manipulated) per affected person decreased from 11 to 6 (Fig 8), representing a relative reduction of 45.5%. In year 2 (rounds 5–8), the median number of sand flea lesions among affected individuals was consistently below 6.

**Fig 9:**
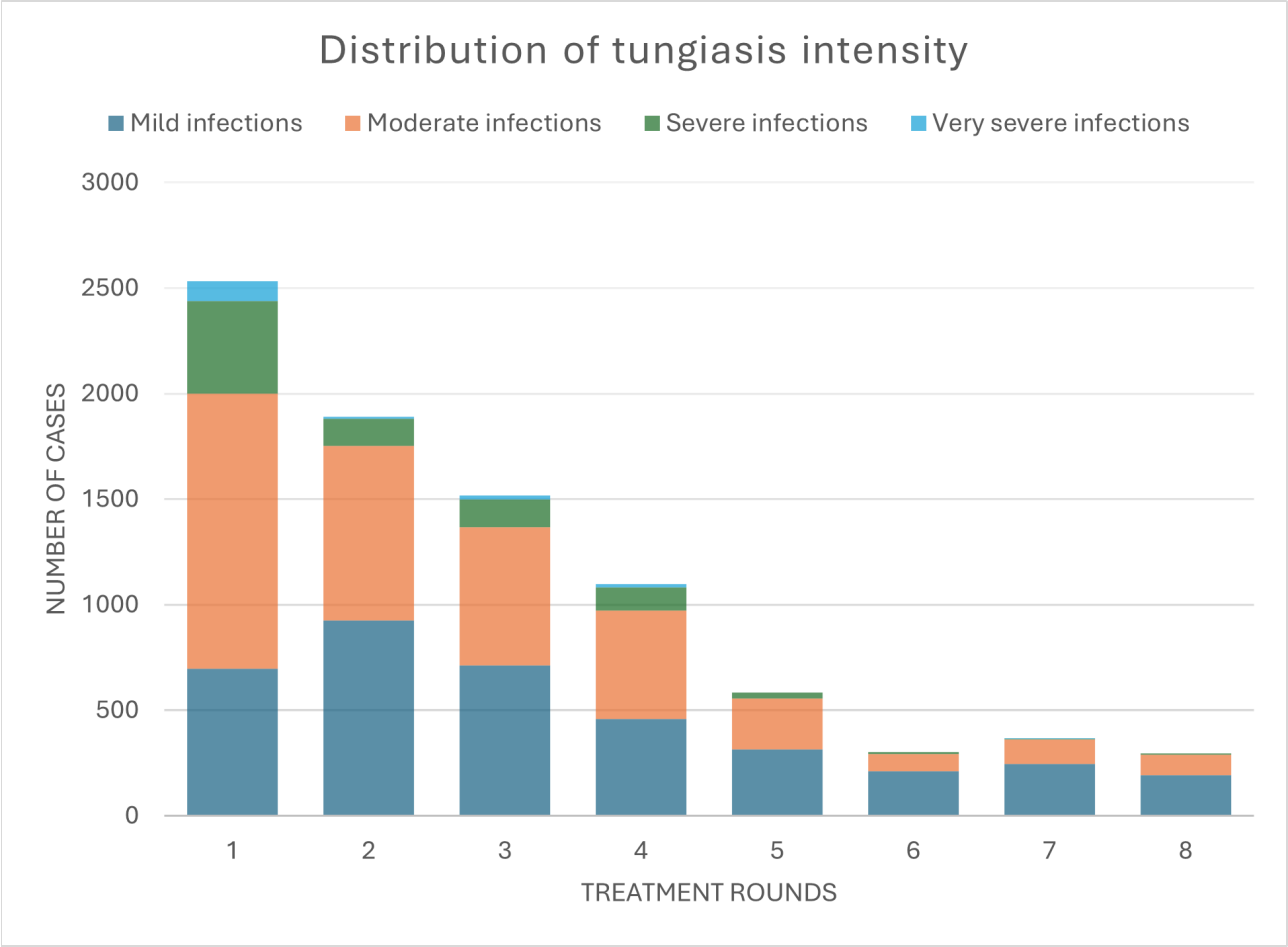
Proportion of mild, moderate, severe, and very severe infections throughout the study period.

While at baseline mild cases represented a minority (27.5%; n = 696/2,534), one year later (round 5), 53.8% of tungiasis cases were mild (n = 315/585), and at the end of the study, 63.8% of the tungiasis cases were mild (n = 191/296) (Fig 9 and S1 Table). The number of very severe cases rapidly diminished from 96 cases in round 1 to 7 cases in round 2, which represents a reduction of 92.7%. In rounds 5, 6, and 8 no very severe cases were found, and in round 7 we recorded one very severe case (Fig 9 and S1 Table).

The total number of tungiasis lesions in the study area was significantly reduced from 58,806 at baseline to 2,027 at the end of the tungiasis control project (Table 5), representing a relative reduction of 96.6%. Manipulated sand flea lesions were reduced from 29,462 to 340 (Table 5), representing a relative reduction of 98.9%.

**Table 5:** Numbers of tungiasis lesions documented in the study population during the eight treatment rounds.

	Treatment round							
Type of lesion^a^	1	2	3	4	5	6	7	8
**Viable**	18970 (32.3%)	6629 (33.3%)	6002 (31.8%)	4656 (31.2%)	1122 (21.7%)	660 (35.1%)	783 (35.3%)	507 (25.0%)
**Dead**	10374 (17.6%)	3459 (17.4%)	3437 (18.2%)	2844 (19.0%)	1198 (23.2%)	803 (42.7%)	916 (41.3%)	1180 (58.2%)
**Manipulated**	29462 (50.1%)	9828 (49.3%)	9428 (50.0%)	7442 (49.8%)	2852 (55.1%)	419 (22.3%)	519 (23.4%)	340 (16.8%)
**Total**	58806 (100%)	19916 (100%)	18867 (100%)	14942 (100%)	5172 (100%)	1882 (100%)	2218 (100%)	2027 (100%)

^a^see material and methods

### Morbidity

**Fig 10:**
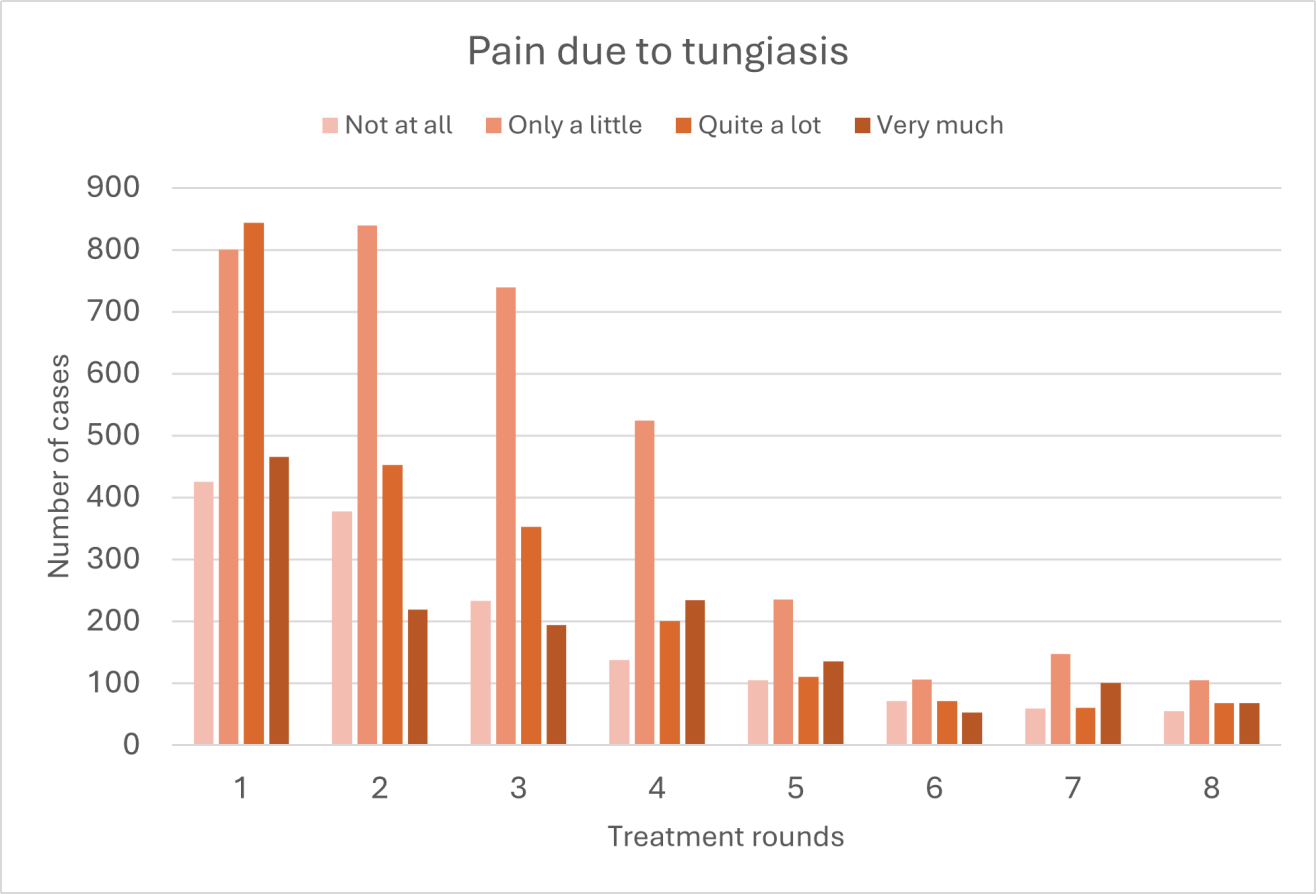
Number of cases reporting different intensities of pain due to tungiasis. S2 Table in the Supplement shows the data including case numbers and percentages.

**Fig 11:**
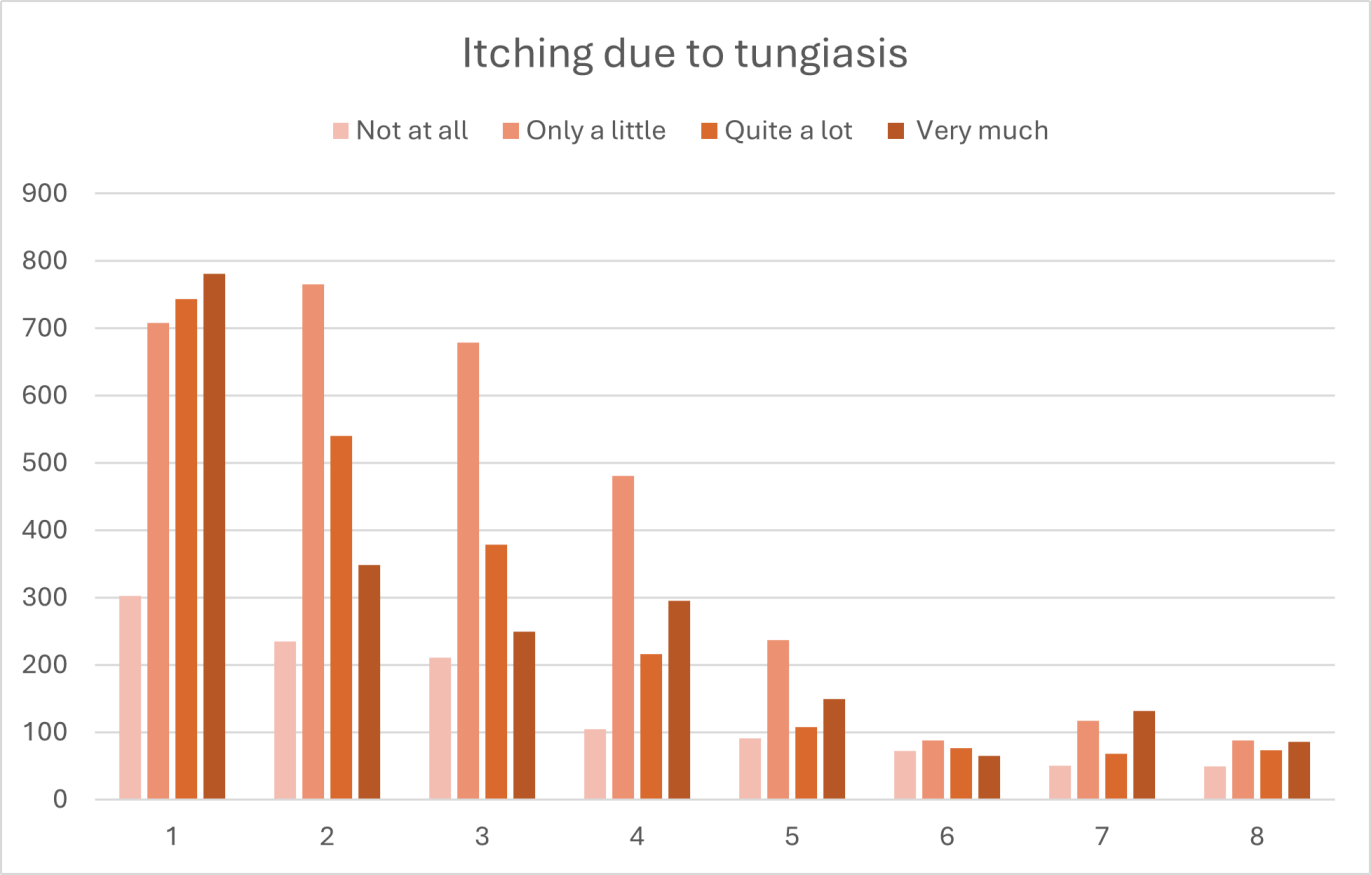
Number of cases reporting different intensities of itching due to tungiasis. S3 Table in the Supplement shows the data including case numbers and percentages.

In line with the reduction of prevalence, tungiasis-related pain, itching, and walking difficulties were reduced significantly throughout the tungiasis control project period. Stronger pain and itching (‘quite a lot’ and ‘very much’) declined more rapidly than weaker pain and itching (‘a little’) (Figs 10 and 11; S2 and S3 Tables).

**Fig 12:**
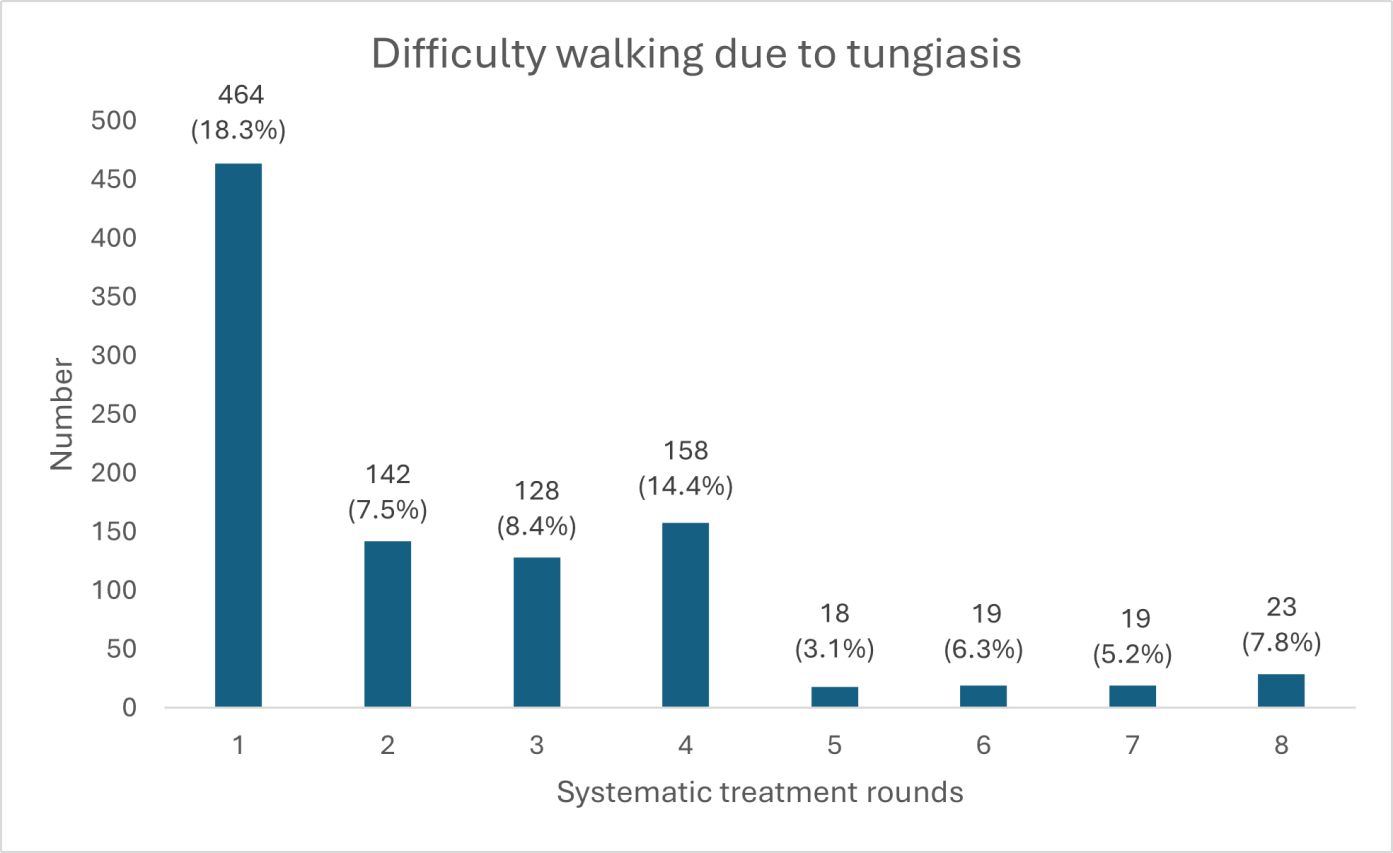
Number of cases reporting difficulty walking due to tungiasis. Percentages relate to the total number of tungiasis cases per diagnosis and treatment round.

The prevalence of tungiasis-related walking difficulties in the community dropped from 11.5% at baseline (n = 464 out of 4,035) to 0.5% in round 8 (n = 27 out of 5,155) (Fig 12). After the first round of treatment, the number of cases with tungiasis-related walking difficulty decreased from 464 to 142, which represents a reduction of 69.4%. When comparing the overall number of cases with tungiasis-related walking difficulty in year 1 (rounds 1–4) with year 2 (rounds 5–8), we saw a decrease of 90.9%, from 892 cases with walking difficulty during year 1 (range = 128–464) to 79 cases with walking difficulty during year 2 (range = 18–23).

### Tungiasis among visitors

In each treatment round, the project team recorded between 5 and 43 visitors from other areas (median = 25). Out of the overall 187 visitors, 74 (39.6%) had tungiasis. Tungiasis prevalence among visitors ranged from 10.5% and 100% between the eight systematic rounds (median = 42.5%). In almost all treatment rounds, tungiasis among visitors was more common than in the overall study population; except for round 5, where tungiasis occurred in 10.5% of visitors (2/19) compared to 15.9% among the residents of the study area.

### Tungiasis in animals

**Fig 13:**
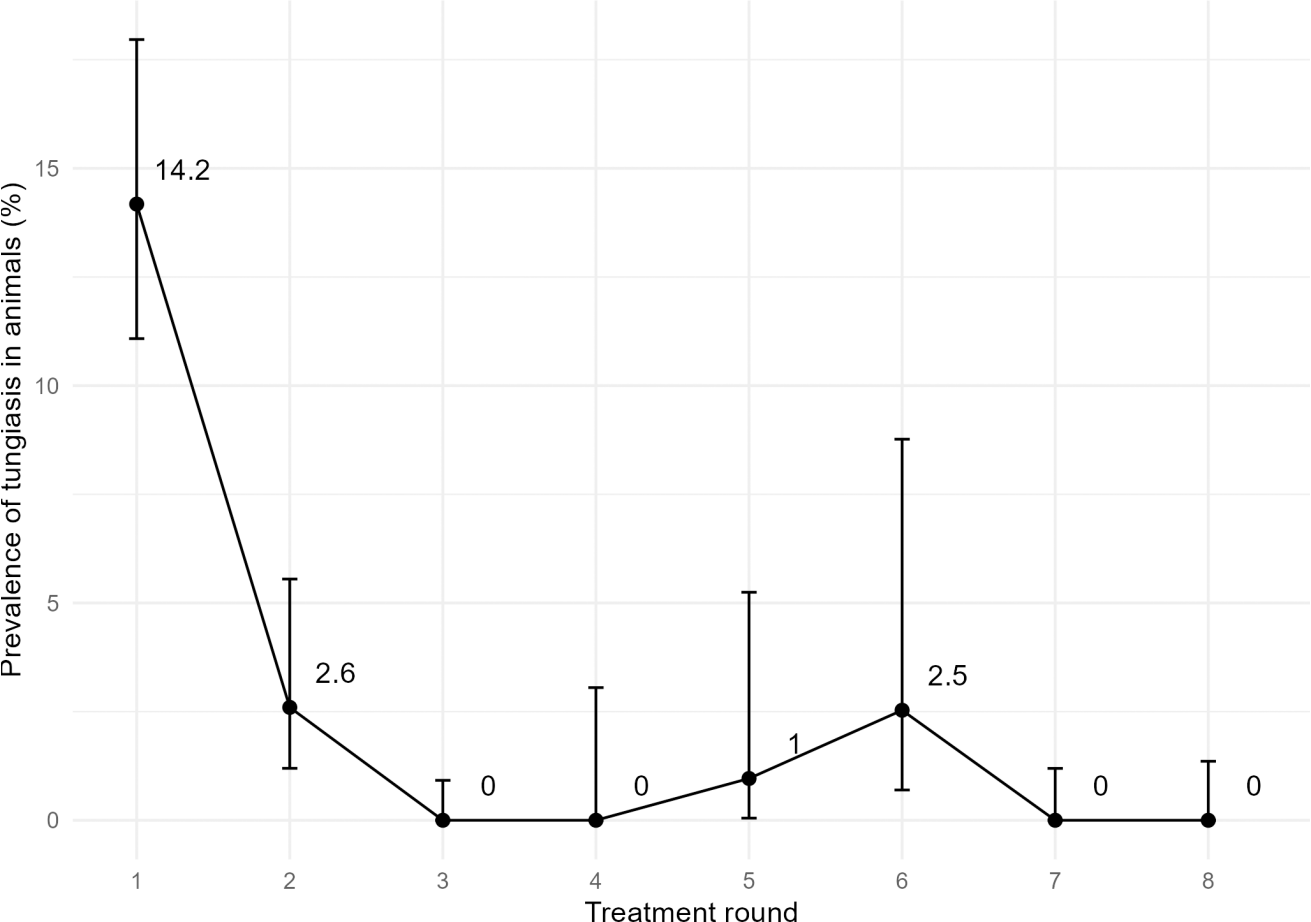
Prevalence of tungiasis among animals present in the study area.

Relative to the number of people included in this study, the number of animals was very low, as livestock was mostly kept far away from the villages due to rampant armed raids in the study area. Throughout the eight rounds of case detection and treatment, 1,942 examinations of domestic animals (excluding poultry) took place in the villages, compared with 35,940 examinations carried out among humans. Numbers of animals found in the study area varied between 414 (round 3) and 79 (round 6), with a median of 255. The animals which were most commonly present during treatment rounds were sheep and goats (median 93.5, range 22–270; and median 86.5, range 22–113, respectively). Less frequently, dogs (median = 8.5, range = 2–92), cats (median 5, range 0–86), and cattle (median 11, range 0–25) were present. Pigs were rarely kept (median 3.5, range 0–19), however, they were disproportionally highly affected with tungiasis. At baseline (detection and treatment round 1), 14.2% (n = 56/395) of all domestic animals in the villages had tungiasis (Fig 13), and 80.0% of pigs (n = 8/10) were affected. By round 2, tungiasis prevalence in animals had fallen to 2.6% (n = 6/231). Only three tungiasis cases in animals were found throughout the remainder of the tungiasis control project period (rounds 3–8); these were one dog in round 5 and two pigs in round 6.

## Discussion

This large-scale community-based study evaluated a One Health project that aimed at reducing the burden of tungiasis in a hyperendemic community, combining treatment of humans and animals with community engagement. In Nigeria, a smaller study in one village showed that preventive environmental measures alone, namely covering house floors with concrete, banning free-roaming pigs, and promoting environmental sanitation and use of footwear, reduced tungiasis prevalence from 45.2% to 21.3% after one year [40]. In comparison, in our study we saw a greater reduction from 62.8% to 15.9% after one year. A study of a 3-month long tungiasis control project from Brazil [54] which combined surgical extraction of sand fleas from humans with animal treatment with ectoparasiticides and spraying of insecticides in houses, reduced the prevalence of tungiasis temporarily from 37% to 10% after one year; however, in the following dry season, the prevalence had reached previous level again. Finally, a 1.5 year-long tungiasis control project among small indigenous villages in the Brazilian Amazon region with baseline prevalence of 8.7% eliminated tungiasis in the community by way of a similarly comprehensive approach to ours, including regular use of topical dimeticone for humans, oral insecticide treatment for dogs, and fumigation of houses [55]. These studies are not directly comparable to ours due to the different settings (e.g., pigs being an important reservoir in the Nigerian village, or the low baseline prevalence among the indigenous group in Brazil). However, in conjunct with our results they suggest that long-term systematic treatment for tungiasis in humans and animals together with community-based prevention measures is superior to environmental measures alone and to short-term interventions.

In addition to the reduction in prevalence, tungiasis intensity decreased and cases were more likely to be mild towards the end of the presented tungiasis control project. The prevalence of very severe infections declined fastest and had almost disappeared in the second year of the project. The decreased tungiasis prevalence led to a considerable reduction of morbidity (pain, itching, and impaired mobility) in our study population. Studies with children in Kenya and Ethiopia showed that tungiasis morbidity significantly reduces quality of life and school performance and increases absenteism [15,23,56]. In one Kenyan study, 86% and 84% of affected children reported sleep disturbances and concentration difficulties, respectively, which were significantly correlated to the intensity of tungiasis and quickly resolved after treatment [15]. Another Kenyan study showed that children with more severe tungiasis experienced lower quality of life than those with mild tungiasis [23]. Moreover, it is known that people with skin NTDs frequently suffer from stigmatisation and social exclusion [21,22,57,58]. In a previous publication we have shown that tungiasis stigma is highly prevalent in our study area in Napak, with people commonly associating tungiasis with being ‘dirty’, ‘lazy’, and ‘irresponsible’ [18]. We thus assume that the humanitarian tungiasis control project has considerably improved the quality of life in the study area, including better ability to work and attend school, less suffering due to reduced morbidity, and less stigmatisation and social exclusion of affected individuals.

Throughout the project period, one parish (Nagule Angolol) had higher tungiasis prevalences than the two other parishes (Naitakwe and Nawaikorot). While the absolute reduction of tungiasis between the start and end of the project was higher in Nagule Angolol than in the other parishes, the relative reduction was lower. This variation could be explained by the fact that villages in Nagule Angolol parish are clustered in a small area where people lived in crowded homesteads, whereas villages in Naitakwe and Nawaikorot parishes were more distributed geographically (see Fig 1). The large decline in tungiasis prevalence and morbidity in all three parishes shows that the comprehensive project design was successful in these different settings.

At baseline, younger children (5–14 years) and the elderly (60 years or older) were most affected with tungiasis, which represents the typical S-shape distribution of age-specific tungiasis prevalence [10]. While tungiasis prevalence decreased rapidly in both children and the elderly and across genders, it is important to highlight that from round 3 until the end of the study, tungiasis prevalence among elderly women remained higher than in any other demographic group. This indicates that tungiasis control projects in similar settings should pay special attention to supporting tungiasis control efforts among elderly women.

In year two of the humanitarian project, the overall tungiasis prevalence reached a plateau at low level (between 5.7% and 7.5% in treatment rounds 6, 7, and 8). This plateau might indicate that a level of morbidity was reached where the population was less motivated to increase their tungiasis control efforts further. Another reason for the stagnating decrease in tungiasis prevalence at the end of year 2 may be the fact that visitors kept re-introducing sand fleas to the villages, as in all systematic rounds (except for round 5) we found higher tungiasis frequencies in visitors compared with residents in the study parishes. A recent field observation of a heavily infected visitor from outside of the project area showed that hundreds of sand flea eggs were expelled and fell to the ground within 10 minutes after the visitor had sat down on a chair (F. Mutebi, H. Feldmeier, personal observation 2023).

In our study, the VTHWs documented that they had independently applied 4,198 interround treatments to community members, compared to 8,342 treatments during the systematic rounds. As embedded sand fleas start shedding eggs about 6 days after penetration of the skin [2], early treatment can prevent the spread of eggs in the environment and the development of severe morbidity. Easy access to tungiasis treatment and continuation of environmental tungiasis control will therefore be key to sustain the success of tungiasis control initiatives in the long term.

Our study was designed as a One Health intervention. Unexpectedly, the number of tungiasis cases that we found in animals was minimal compared with tungiasis cases in humans (65 and 12,540, respectively). Only few animals were present in the villages. Among these, tungiasis was rare, with a prevalence of 14.2% at baseline and only single cases thereafter. This finding differs from a study in Bugiri, Eastern Uganda, where prevalences of animal and human tungiasis were similar (median of 22% in animals and 28% in humans) [41]. Studies from rural Nigeria and Uganda identified pigs as the most relevant tungiasis reservoirs [41,43] while a study in an impoverished urban settlement in Brazil found high tungiasis prevalences in cats and dogs [42]. Indeed, at baseline, 80% of pigs and 24% of dogs in our study area were affected with tungiasis and hence represent a small but relevant reservoir for tungiasis [10]. The low prevalence of animal tungiasis in our study area may be explained with the facts that a) pigs were rarely kept, b) most sheep, goats, and cows were shepherded far away from the villages and c) cats and dogs were generally not allowed in the houses and were often moving with animal herds far away from households.

Our finding that 50% of tungiasis lesions at baseline had been manipulated underscores our previously published finding that the most commonly practiced tungiasis treatment measure was manual extraction of sand fleas with unsterile pins, thorns, needles, or razor blades which were often shared within a household and sometimes within the community [18,28]. This painful and dangerous practice is a sign of sheer desperation and is widely used among endemic communities [4,27,28]. Manual extraction can cause physical and psychological trauma and facilitates the transmission of bacterial and viral infections, predisposing to septicaemia, tetanus, and hepatitis B and C [1,16,28]. As a result of the presented tungiasis control project, the number of manipulated lesions was reduced by 98.9% from 29,462 at baseline to 340 at the end of the study. We estimate that several hundred thousand painful extractions and open wounds have been prevented over the two years of the project, hence significantly decreasing the risk of potentially life-threatening infections in the study population. When safe and effective treatment is made available, the application of hazardous substances like tobacco, kerosene, and motor oil [18,29,39] as well as mechanical extraction of sand fleas should become obsolete practices.

A limitation of this study is that our team could not assess systematically to what degree the proposed hygiene measures and smearing of the floors were followed in the community, due to logistical constraints and because hygiene monitoring could easily have been experienced as intrusive and inappropriate by the local population. Furthermore, as this was not a randomized controlled trial, we can only assume that the humanitarian project contributed to the reduction of tungiasis prevalence and intensity. Due to ethical implications, it was not possible to assess tungiasis in a control group without offering treatment and information about tungiasis prevention. However, it is highly unlikely that other factors led to this dramatic change, since tungiasis was a longstanding problem in the area and at the end of the study the prevalence was still high in neighbouring parishes (personal observation, F. Mutebi, G.M. Mukone, H. Feldmeier). Another limitation is that the interround treatments, which were independently administered by VTHWs without supervision, might not have been documented as reliably as the systematic round treatments, so that the total number of treatments may have been underestimated. It should further be noted that, as part of a separate tungiasis school project carried out by IFOTRODE (March to December 2022), the VTHWs treated 190 tungiasis cases among children from our study area in local schools with the dimeticone formula.

Regarding sustainability of the success of the humanitarian project, it is conceivable that tungiasis prevalence would increase again if effective tungiasis control measures are not made consistently available in the future, as tungiasis could not be completely eliminated in our study area and is still highly endemic in neighbouring areas. While IFOTRODE was able to supply the VTHWs in the study area with the dimeticone oil formula for another two years following the end of the study, public health services need to sustain tungiasis control in the area in the long term. The addition of NYDA to Uganda’s list of essential medicines in 2023 [51] and the development of a national treatment guideline [52] represent milestones in tungiasis control in Uganda and Africa in general.

## Conclusion

Our results show that regular community-based treatment of tungiasis cases among humans and animals with dimeticone oil formula, coupled with community engagement and health education, can effectively reduce tungiasis prevalence, intensity, and morbidity to very low levels, even in a hyperendemic area where people live in extreme poverty.

We recommend that similar community-based tungiasis control projects as well as education about tungiasis control among affected communities, healthcare professionals, and political stakeholders are implemented in other endemic regions.

## Supporting information

S1 Fig Tungiasis prevalence stratified by age group and gender per treatment round. (DOCX)

S1 Table Number and proportion of different categories of infection intensity among detected cases during treatment rounds. (DOCX)

S2 Table Number and proportion of different categories of pain intensity among detected cases during treatment rounds. (DOCX)

S3 Table Number and proportion of different categories of itching intensity among detected cases during treatment rounds. (DOCX)
