# Supplementary figures and images for: Reduction of tungiasis prevalence, intensity, and morbidity during a two-year long community-based tungiasis control project in a hyperendemic region in Karamoja, Uganda
Source: PLoS Negl Trop Dis. 2025 Jun 5;19(6):e0013149. doi: 10.1371/journal.pntd.0013149 (PMC12173417; doi:10.1371/journal.pntd.0013149)

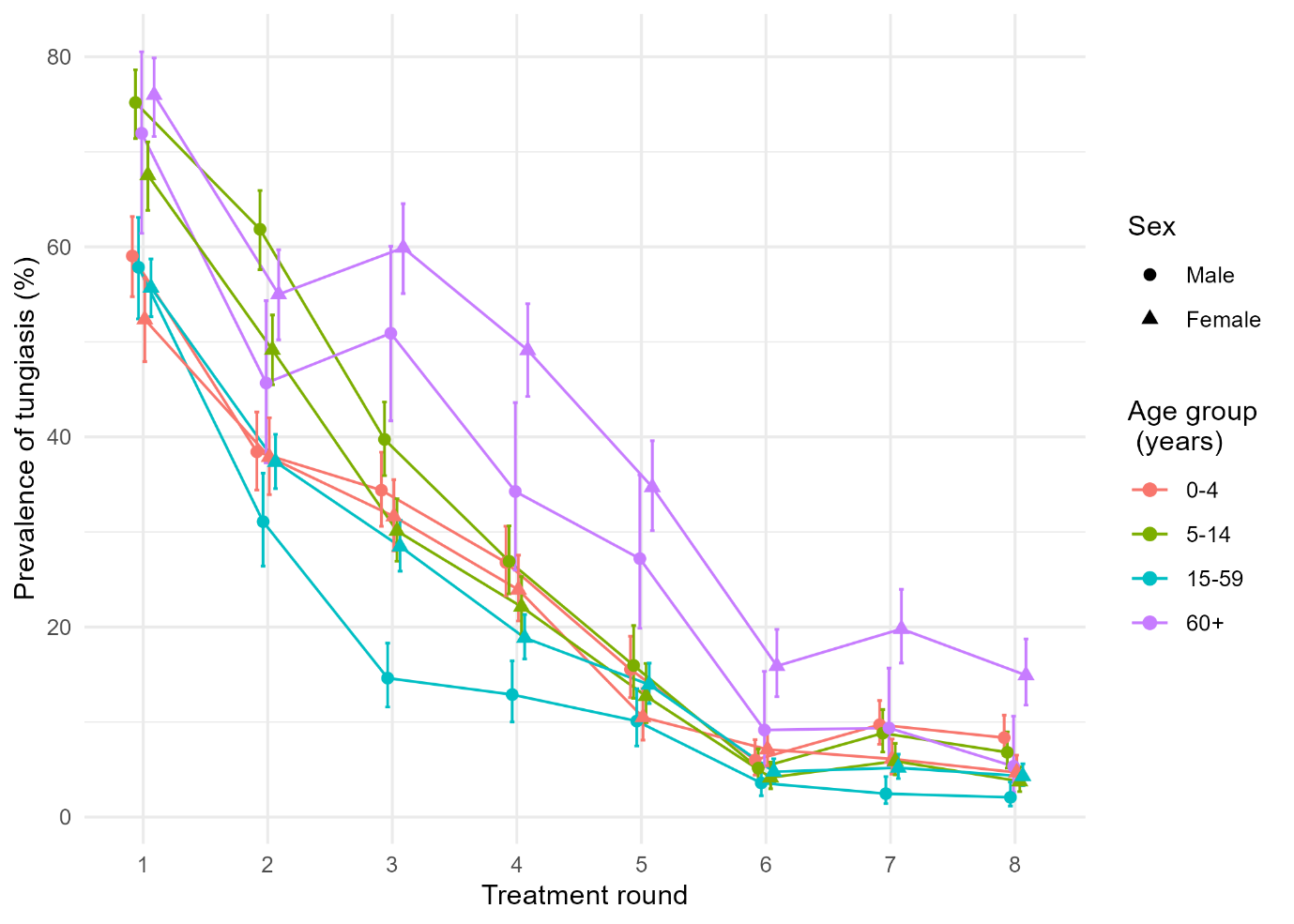


S1 Fig: Tungiasis prevalence stratified by age group and gender per treatment round

Supplement: S1 Fig — (DOCX) [file pntd.0013149.s001.docx]
