# Supplementary material for: Reduction of tungiasis prevalence, intensity, and morbidity during a two-year long community-based tungiasis control project in a hyperendemic region in Karamoja, Uganda
Source: PLoS Negl Trop Dis. 2025 Jun 5;19(6):e0013149. doi: 10.1371/journal.pntd.0013149 (PMC12173417; doi:10.1371/journal.pntd.0013149)
Supplement: S1 Table — (DOCX) [file pntd.0013149.s002.docx]

Tungiasis infection intensity

| Treatment round | Mild infections | Moderate infections | Severe infections | Very severe infections | Total |
| --- | --- | --- | --- | --- | --- |
| 1 | 696 (27.5%) | 1304 (51.5%) | 438 (17.3%) | 96 (3.8%) | 2534 (100%) |
| 2 | 925 (49.0%) | 828 (43.8%) | 129 (6.8%) | 7 (0.4%) | 1889 (100%) |
| 3 | 713 (46.9%) | 654 (43.1%) | 133 (8.8%) | 19 (1.3%) | 1519 (100%) |
| 4 | 460 (41.9%) | 514 (46.9%) | 109 (9.9%) | 14 (1.3%) | 1097 (100%) |
| 5 | 315 (53.8%) | 240 (41.0%) | 30 (5.1%) | 0 (0.0%) | 585 (100%) |
| 6 | 210 (69.8%) | 82 (27.2%) | 9 (3.0%) | 0 (0.0%) | 301 (100%) |
| 7 | 245 (66.8%) | 115 (31.3%) | 6 (1.6%) | 1 (0.3%) | 367 (100%) |
| 8 | 191 (64.5%) | 98 (33.1%) | 7 (2.4%) | 0 (0.0%) | 296 (100%) |

S1 Table: Number and proportion of different categories of infection intensity among detected cases during treatment rounds.
