# Supplementary material for: Reduction of tungiasis prevalence, intensity, and morbidity during a two-year long community-based tungiasis control project in a hyperendemic region in Karamoja, Uganda
Source: PLoS Negl Trop Dis. 2025 Jun 5;19(6):e0013149. doi: 10.1371/journal.pntd.0013149 (PMC12173417; doi:10.1371/journal.pntd.0013149)
Supplement: S2 Table — (DOCX) [file pntd.0013149.s003.docx]

Pain

| Treatment round | Not at all | Only a little | Quite a lot | Very much | Total |
| --- | --- | --- | --- | --- | --- |
| 1 | 425 (16.8%) | 800 (31.6%) | 843 (33.3%) | 466 (18.4%) | 2534 (100%) |
| 2 | 378 (20.0%) | 839 (44.4%) | 453 (23.9%) | 219 (11.6%) | 1889 (100%) |
| 3 | 233 (15.3%) | 739 (48.7%) | 353 (23.2%) | 194 (12.8%) | 1519 (100%) |
| 4 | 138 (12.6%) | 524 (47.8%) | 201 (18.3%) | 234 (21.3%) | 1097 (100%) |
| 5 | 105 (17.9%) | 235 (40.2) | 110 (18.8%) | 135 (21.3%) | 585 (100%) |
| 6 | 71 (23.6%) | 106 (35.2%) | 71 (23.6%) | 53 (17.6%) | 301 (100%) |
| 7 | 59 (16.1%) | 147 (40.1%) | 60 (16.3%) | 101 (27.5%) | 367 (100%) |
| 8 | 55 (18.6%) | 105 (35.5%) | 68 (23.0%) | 68 (23.0%) | 296 (100%) |

S2 Table: Number and proportion of different categories of pain intensity among detected cases during treatment rounds.
