# Supplementary material for: Reduction of tungiasis prevalence, intensity, and morbidity during a two-year long community-based tungiasis control project in a hyperendemic region in Karamoja, Uganda
Source: PLoS Negl Trop Dis. 2025 Jun 5;19(6):e0013149. doi: 10.1371/journal.pntd.0013149 (PMC12173417; doi:10.1371/journal.pntd.0013149)
Supplement: S3 Table — (DOCX) [file pntd.0013149.s004.docx]

Itching

| Treatment round | Not at all | Only a little | Quite a lot | Very much | Total |
| --- | --- | --- | --- | --- | --- |
| 1 | 303 (11.9%) | 708 (27.9%) | 742 (29.3%) | 781 (30.8%) | 2534 (100%) |
| 2 | 235 (12.4%) | 765 (40.5%) | 540 (28.6%) | 349 (18.5%) | 1889 (100%) |
| 3 | 211 (13.9%) | 679 (44.7%) | 379 (25.0%) | 250 (16.5%) | 1519 (100%) |
| 4 | 105 (9.6%) | 481 (43.8%) | 216 (19.7%) | 295 (26.9%) | 1097 (100%) |
| 5 | 91 (15.6%) | 237 (40.5%) | 108 (18.5%) | 149 (25.5%) | 585 (100%) |
| 6 | 72 (23.9%) | 88 (29.2%) | 76 (25.2%) | 65 (21.6%) | 301 (100%) |
| 7 | 50 (13.6%) | 117 (31.9%) | 68 (18.5%) | 132 (36.0%) | 367 (100%) |
| 8 | 49 (16.6%) | 88 (29.7%) | 73 (24.7%) | 86 (29.1%) | 296 (100%) |

S3 Table: Number and proportion of different categories of itching intensity among detected cases during treatment rounds.
